# Expanding organofluorine chemical space: the design of chiral fluorinated isosteres enabled by I(i)/I(iii) catalysis†

**DOI:** 10.1039/d1sc02880d

**Published:** 2021-06-29

**Authors:** Stephanie Meyer, Joel Häfliger, Ryan Gilmour

**Affiliations:** Organisch-Chemisches Institut, Westfälische Wilhelms-Universität Münster Correnstraße 36 48149 Münster Germany ryan.gilmour@uni-muenster.de

## Abstract

Short aliphatic groups are prevalent in bioactive small molecules and play an essential role in regulating physicochemistry and molecular recognition phenomena. Delineating their biological origins and significance have resulted in landmark developments in synthetic organic chemistry: Arigoni's venerable synthesis of the chiral methyl group is a personal favourite. Whilst radioisotopes allow the steric footprint of the native group to be preserved, this strategy was never intended for therapeutic chemotype development. In contrast, leveraging H → F bioisosterism provides scope to complement the chiral, radioactive bioisostere portfolio and to reach unexplored areas of chiral chemical space for small molecule drug discovery. Accelerated by advances in I(i)/I(iii) catalysis, the current arsenal of achiral 2D and 3D drug discovery modules is rapidly expanding to include chiral units with unprecedented topologies and van der Waals volumes. This *Perspective* surveys key developments in the design and synthesis of short multi*vicinal* fluoroalkanes under the auspices of main group catalysis paradigms.

## Abstract 2

Short aliphatic groups are prevalent in bioactive small molecules and play an essential role in regulating physicochemistry and molecular recognition phenomena.

## Graphical abstract

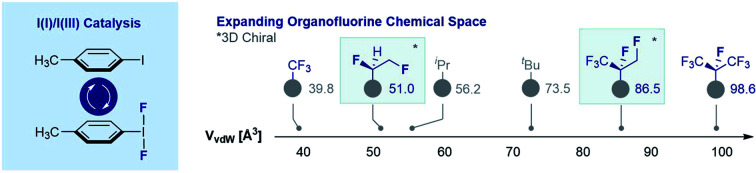

## 1. Introduction

**Fig. 1:**
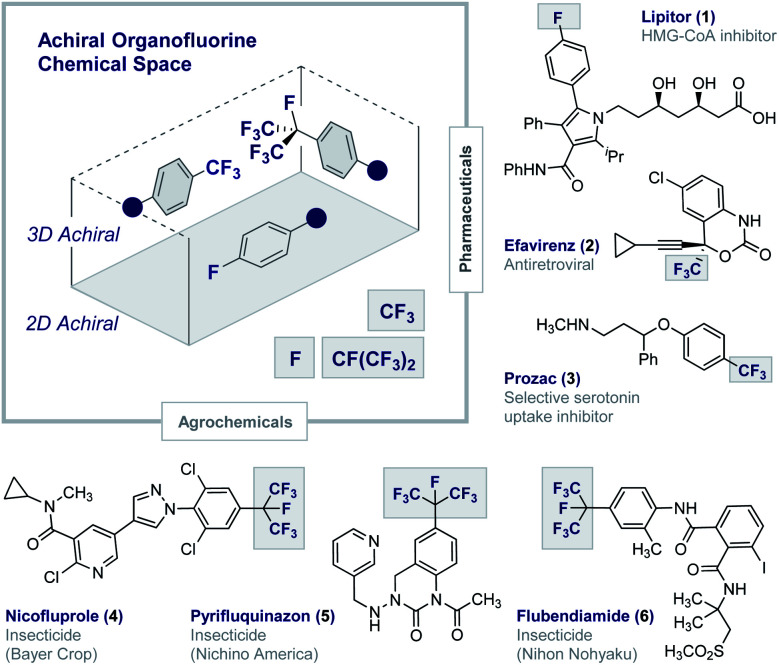
Achiral organofluorine chemical space. Selected examples of blockbuster drugs and agrochemicals containing achiral fluorinated motifs (**1–6**).

Fluorinated architectures traverse the functional small molecule landscape,^1^ where they manifest themselves in blockbuster drugs (**1–3**),^2^ essential agrochemicals (**4–6**)^3^ (Fig. 1) and high-performance materials such as Teflon®.^4^ Ubiquitous in modern society, fluorinated motifs continue to feature in the vanguard of focussed molecular design strategies^5^ with short perfluoroalkyl groups such as CF_3_ and CF(CF_3_)_2_ now enjoying “*privileged*” status.^6,7^ In a reductionist sense, the functional diversity of fluorinated materials can be attributed to the physicochemical consequences of C(sp^2^/sp^3^)-H^δ+^ → C(sp^2^/sp^3^)-F^δ−^ structural editing^8^ and the new regions of chemical space that result.^9^ The (stereo)electronic impact of this (bio)isosterism appears subtle but, when appropriately leveraged, can induce counterintuitive conformational behaviour,^10^ elicit novel molecular recognition modes^11^ and augment stability.^1,2,5^ Whilst this latter consequence of fluorination has been widely lauded as a triumph in bioactive small molecule discovery, it has obvious environmental consequences.^12^ This is unsurprising given the conspicuous dearth of fluorinated natural products^13^ and, by extension, regulatory enzymes to facilitate the construction and degradation of this class of organohalogens.^14^ Reconciling the benefits of short, fluorinated motifs as essential modulators of health and development, with environmental considerations, continues to aggravate this complex relationship. This juxtaposition provides a powerful impetus to explore new areas of organofluorine chemical space to expand the current portfolio of drug and agrochemical discovery modules. Augmenting the current arsenal of achiral 2D and 3D motifs to include chiral 3D topologies will open up a wealth of opportunities,^15^ and simultaneously reduce dependence on perfluorocarbon moieties: this may allow existing degradative enzymes to be harnessed and thus mitigate environmental accumulation.^16^ This personal *Perspective* reflects on the possible motivating factors that have led to a surge of interest in the generation of short, chiral fluorinated groups and highlights the important role of I(i)/I(iii) catalysis as an enabling technology in this arena.

## 2. Short aliphatic groups in (bio)-organic chemistry

### 2.1 Radioisotopes to stable isotopes

**Fig. 2:**
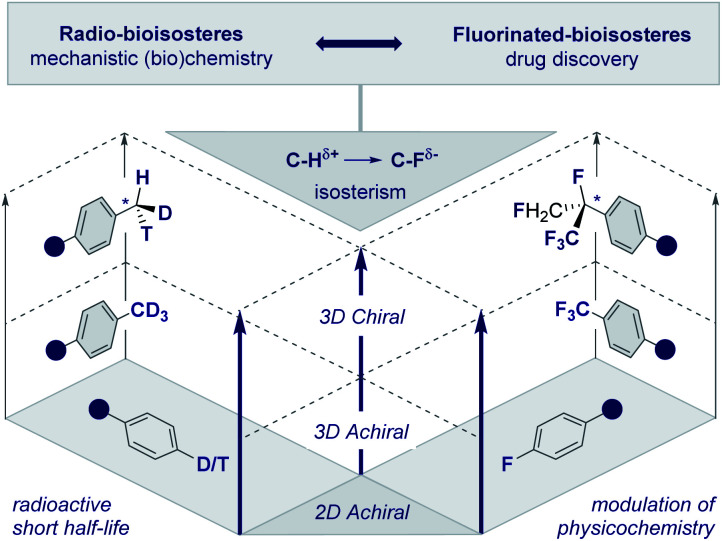
Radio- and fluorinated-bioisosteres: expanding achiral 2D and 3D motifs into chiral 3D chemical space.

The frequency with which simple methyl groups are encountered in the natural product repertoire mirrors the success of its electronic antipode (CF_3_) in contemporary drug discovery. However, striking disparities in the stability of the respective isotopes of H and F render the development of a chiral CF_3_ group improbable. In the case of the parent methyl group, it is possible to exploit the three natural isotopes of hydrogen (^1^H, ^2^H and ^3^H) to generate a stereogenic center and this has been instrumental in the course of mechanistic enzymology (Fig. 2, left, the chiral methyl group).^17^ In addition, deuterium is regularly leveraged in drug discovery to delineate pharmacokinetic parameters^18^ and is now a key feature of deutetrabenazine (Austedo®) to treat Huntington's disease.^19^ Although fluorine has a plethora of known isotopes, it is practically and synthetically implausible to translate this into a “chiral” CF_3_ group. This provides an opportunity for creative endeavour in conceiving and evaluating new chemical entities based on short aliphatic groups (C_1_–C_10_). Inspiration can be gleaned in abundance from the bioactive small molecule repository (*vide infra*), where both linear and branched groups (*e.g.*^*t*^Bu in ginkgolide B) are well represented. This will ultimately result in an array of new chiral entities with distinct properties that will complement the aliphatic series.

### 2.2 Expanding organofluorine chemistry beyond achiral 2D and 3D chemical space

**Fig. 3:**
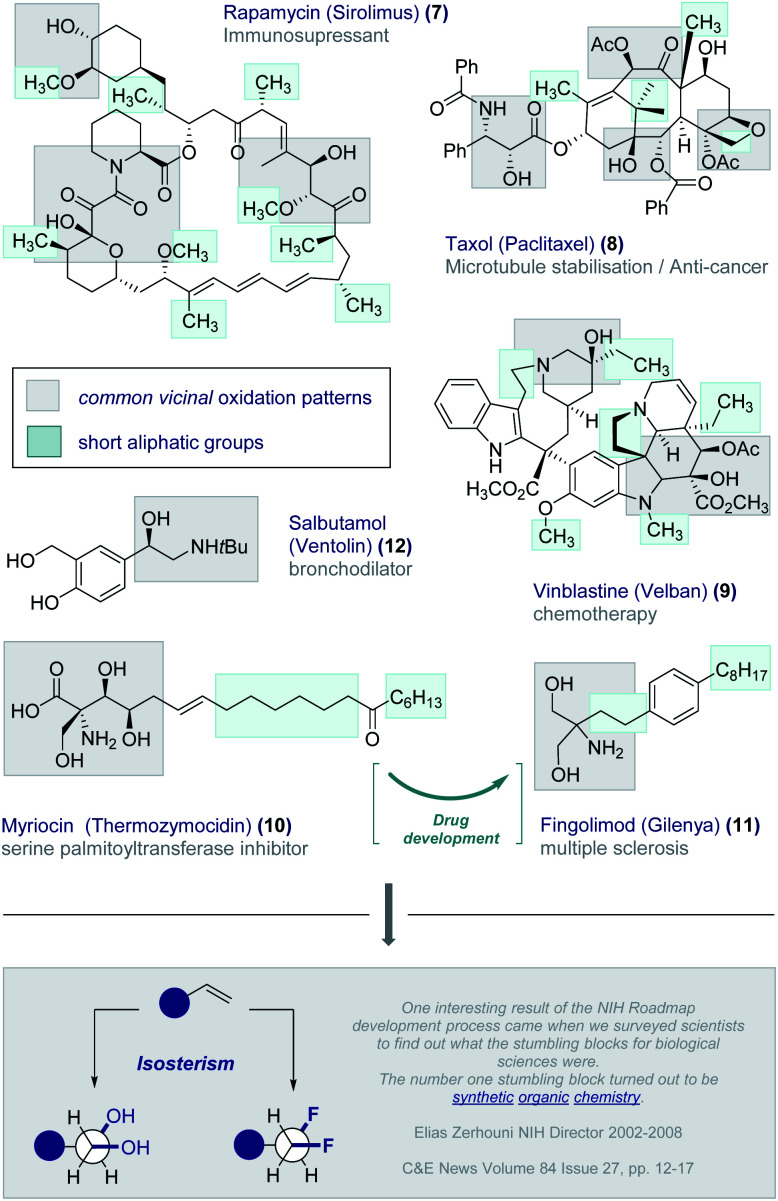
*Vicinal* oxidation patterns and short aliphatic groups in bioactive molecules.

In our quest to design short, chiral fluorine-containing groups, and having disregarded isotope discrimination blueprints from the outset, the formal oxidation of a C_2_ fragment was an appealing starting point. *Vicinal* oxidation is pervasive across the bioactive small molecule spectrum and is intimately involved in orchestrating structure–function interplay.^20^ Examples abound and include the immunosuppressant Rapamycin (Sirolimus) (**7**), the anti-tumour agents Taxol (Paclitaxel) (**8**) and Vinblastine (Velban) (**9**), and the serine palmitoyltransferase inhibitor Myriocin (Thermozymocidin) (**10**) (Fig. 3). It is pertinent to note that this natural product provided the inspiration for Fingolimod (Gilenya®) (**11**) to treat relapsing remitting multiple sclerosis.^21^ A conspicuous feature of these bioactive molecules is the presence of both short alkyl fragments and *vicinal* oxidation patterns. Indeed, this latter feature commonly occurs in the low molecular weight APIs such as the bronchodilator Salbutamol (Ventolin®) (**12**).^22^ It was envisaged that integrating these two common structural features in the development of a short, chiral fluorinated group would also provide a much-needed solution to generating a bioisostere of the *vicinal* diol motif. Whilst OH → F bioisosterism is well established,^6^*vicinal* difluorination strategies are comparatively underdeveloped. This is noteworthy given the interest in halogenated natural products containing contiguous halogen centres,^23^ including the prominent synthesis of a fluorinated analogue of the sulfolid danicalipin A by Carreira and co-workers.^24^

The conspicuous absence of selective *vicinal* difluorination protocols is in stark contrast with the prominence of fluorination patterns in the drug discovery process. This may reflect a limitation in synthetic organic chemistry as opposed to a lack of suitability as drug discovery modules. This echoes the sentiments expressed by former NIH Director Zerhouni that *“One interesting result of the NIH Roadmap development process came when we surveyed scientists to find out what the stumbling blocks for biological sciences were. The number one stumbling block turned out to be synthetic organic chemistry.“*^25^ As Seebach commented in his celebrated essay “*Organic Chemistry: Where Next*?”,^26^ “*molecular function and activity now occupy centre stage*”: realising this objective will require practitioners of organic chemistry to address deficiencies in the synthesis arsenal, such as the fundamental task of adding molecular fluorine across an alkene in a mild and selective manner. Achieving parity with *vicinal* chlorination and bromination, and expanding the protocol to enable the synthesis of telescoped multi*vicinal* fluoroalkanes requires innovative solutions. This latter aspect is particularly urgent given the potential of these materials in the life sciences and materials fields (*vide infra*).

### 2.3 Multi*vicinal* fluoroalkanes (**C2–C6**)

**Fig. 4:**
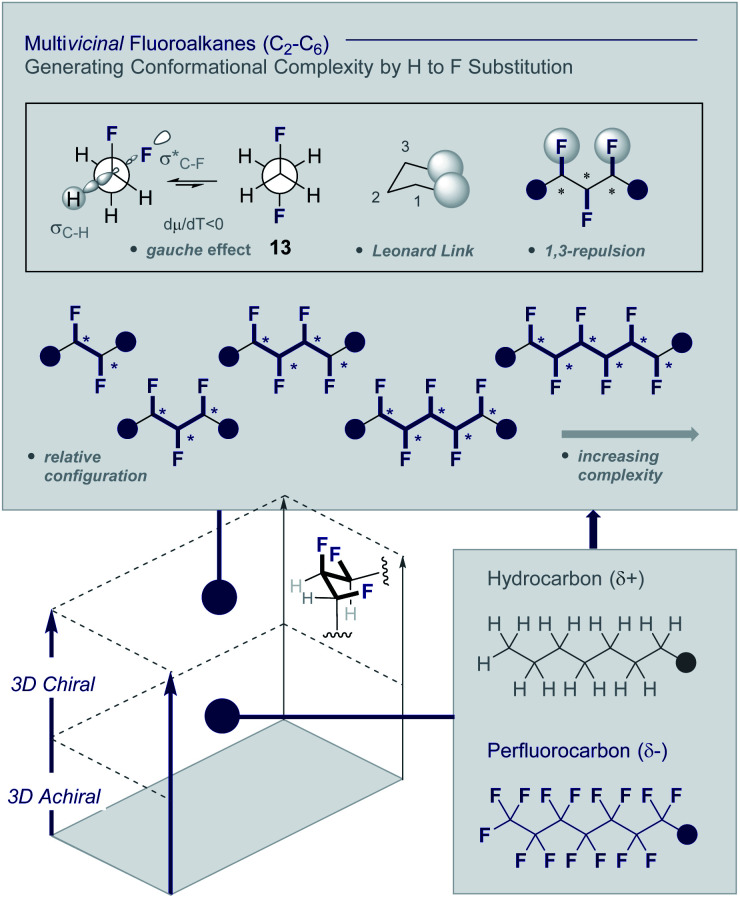
Multi*vicinal* fluoroalkanes: merging hydrocarbons with perfluorocarbons to generate conformational diversity (* denotes a stereogenic centre).

Multi*vicinal* fluoroalkanes are an evolving class of hydrocarbon/polyfluorocarbon hybrids that are composed of repeating CHF units. The simplest member of this organohalogen class may be accessed by the programmed addition of fluorine across an alkene unit (Fig. 4).^27^

**Fig. 5:**
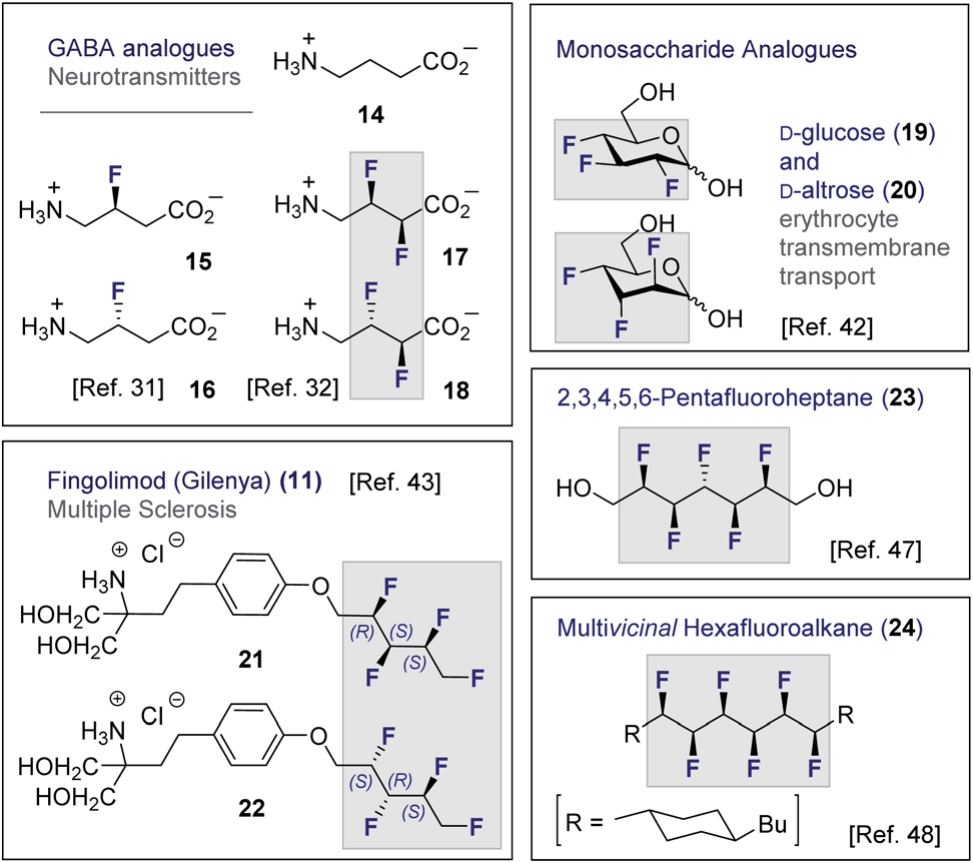
Selected examples of short, multi*vicinal* fluoroalkane groups in functional small molecule design.

Although fluorine has a small van der Waals radius, it is highly electronegative and therefore the inclusion of multiple C (sp^3^)-F bonds along a carbon chain regulates conformation and physicochemistry. The relative configuration of the system gives rise to distinct topologies that manifest stabilising, second-order hyperconjugative interactions (σ_CH_ → σ_CF_*; the venerable stereoelectronic *gauche* effect in 1,2-difluoroethane **13**)^1,10^ and mitigate 1,3-repulsion.^28,29^ The latter acyclic conformational control aspect becomes particularly dominant in systems where *n* ≥3 due to formation of the venerable *Leonard Link*.^28,30^ Since each carbon homologation enables the generation of 2^*n*^ stereoisomers (for *n* homologated carbons), these materials have the potential to significantly expand organofluorine chemical space (**13**): this necessarily requires the development of effective, stereocontrolled methods to facilitate synthesis. Pioneering studies, most notably by O'Hagan and co-workers,^27*b*^ have culminated in the synthesis and physicochemical evaluation of several multi*vicinal* fluoroalkane scaffolds. These elegant routes leverage (asymmetric) oxidation/stereospecific fluorodeoxygenation protocols to efficiently access the target scaffolds of interest. Applications range from the design of peptide mimics to regulate conformation (Fig. 5), through to the introduction of novel liquid crystals. Pertinent examples include the strategic use of fluorination to explore conformational effects in the neurotransmitter GABA (**14**, **15**/**16** and **17**/**18**),^31,32^ to compare the *erythro*- and *threo*-diastereoisomers of 1,2-difluorodiphenylethanes and 2,3-difluorosuccinic acid derivatives,^33^ and to regulate the conformation of simple peptides.^34–37^ Augmentation to the *vicinal* α,β,γ-trifluoro array has been achieved and applied to the synthesis of peptides,^38–40^ liquid crystals^41^ and unnatural monosaccharides (*e.g.***19** and **20**).^42^ More recently, the (terminal) tetrafluoro structural unit has been explored in analogues of the multiple sclerosis drug Gilenya® (**11** – Fig. 3, **21** and **22**).^43^ Remarkably, the O'Hagan laboratory have also reported synthesis routes to (internal) *vicinal* tetrafluoro-,^44–46^ pentafluoro- (**23**)^47^ and hexafluoro-^48^ motifs (**24**).

**Fig. 6:**
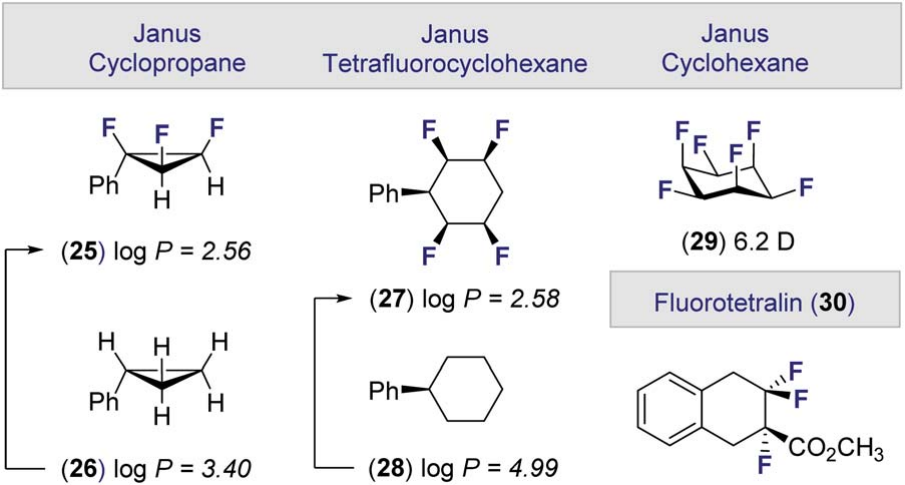
Selected examples of cyclic multi*vicinal* fluoroalkanes by O'Hagan and co-workers.

These advances in the stereocontrolled synthesis of linear multi*vicinal* fluoroalkanes have been complemented by equally impressive synthesis campaigns to generate cyclic motifs (Fig. 6). Many of these materials, in which the fluorine atoms are in an all-*syn* relationship, display significantly lower log *P* values than the parent hydrocarbon. Examples of these facially polarised “*Janus*” motifs include the all-*cis* 1,2,3-trifluorocyclopropane **25** (*cf*. **26**)^49^ and the tetrafluorocyclohexane **27** (*cf*. **28**).^50^ It is interesting to note that the all-*cis* hexafluorocyclohexane **29** has the highest calculated dipole of any organic molecule (6.2 D).^51^ These materials, together with selectively fluorinated tetralins (**30**),^52^ hold great potential as drug discovery modules owing to their well-defined conformations and physicochemical profiles.^53^

## 3. Catalysis-based strategies to access short (≤C_6_), chiral fragments

**Fig. 7:**
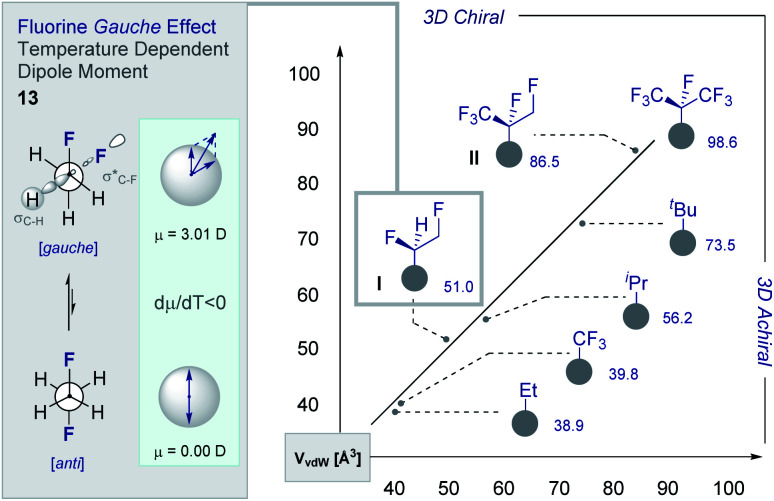
Calculated van der Waals radii [Å^3^] of short aliphatic and fluorinated aliphatic groups. Inset: the stereoelectronic *gauche* effect and the temperature-dependent dipole moment intrinsic to 1,2-difluoroethane.

The structural and physicochemical diversity intrinsic to multi*vicinal* fluoroalkanes is expansive and renders this class of materials valuable in expanding (chiral) organofluorine chemical space. This is evident from a comparative analysis of the van der Waals radii [Å^3^] of common short alkyl groups with their selectively fluorinated counterparts (Fig. 7).^54,55^ Not only are the two partially fluorinated groups (**I** and **II**) chiral, they have volumes and 3D topologies that are complementary to structurally related aliphatic groups. Furthermore, the inclusion of short, chiral fluorinated moieties in the drug discovery portfolio redresses the current bias that favours isotropic groups over anisotropic fragments. The simplest member of the multi*vicinal* fluoroalkane family is structure **I**, which is based on 1,2-difluoroethane (**13**). These structures are intriguing on account of the stabilising hyperconjugative interactions that give rise to the iconic *gauche* conformation.^1,8*b*,10*b*,*c*^ This phenomenon can be rationalised by invoking stabilising σ_C–H_ → σ*_C–F_ interactions and gives rise to a temperature-dependent dipole moment (d*μ*/d*T* <0) (Fig. 6, left). The *gauche* effect is a unique feature of fluorinated materials and is not observed in the corresponding chloro- or bromo-systems due to overriding repulsion.^56^ Collectively, these structural features are compelling arguments for the development of efficient strategies to allow small chiral groups to be assessed in the context of contemporary drug discovery.

### 3.1 Catalysis-based *vicinal* difluorination of alkenes

**Fig. 8:**
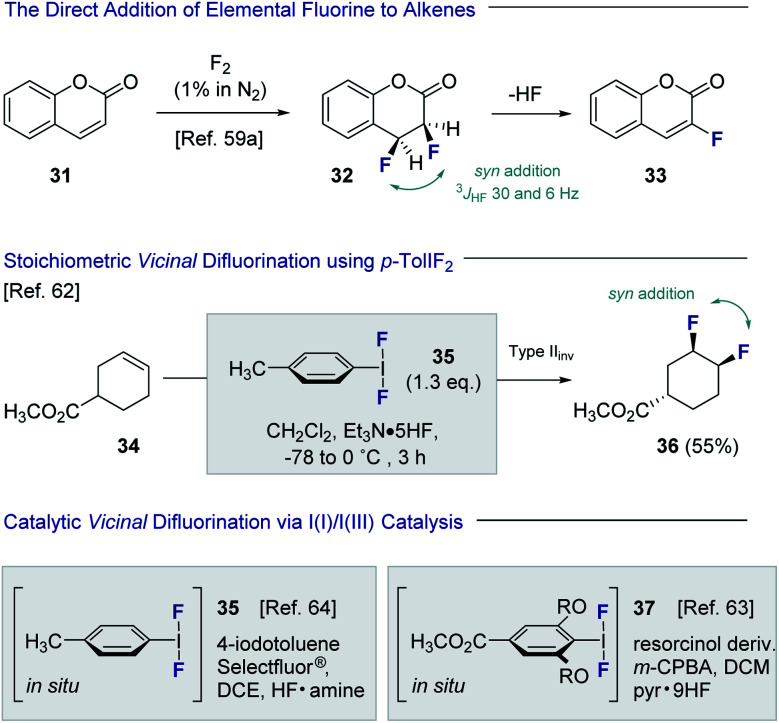
The direct *vicinal* difluorination of alkenes *via* I(i)/I(iii) catalysis.

Despite the popularity of fluorine bioisosterism in medicinal chemistry, and the notable advances in fluorination technologies that this has inspired,^57^ the catalytic, stereoselective *vicinal* fluorination of alkenes is comparatively under-developed.^58^ Direct fluorination using gaseous F_2_ in a carrier gas been reported by Rozen and Brand,^59,60^ but this approach presents safety and operational challenges for non-specialists that must be addressed (Fig. 8). As is evident from the conversion of coumarin **31** to product **32**, the *vicinal* difluorination proceeds in a *syn*-selective fashion as was determined by coupling constant analysis (^3^*J*_HF_ = 30 and 6 Hz). As a consequence, HF elimination occurs to generate the fluorinated coumarin **33**. Tius has demonstrated that XeF_2_ enables the 1,2-difluorination of alkenes, thereby mitigating the safety concerns associated with handling strongly oxidising fluorine gas. Despite the operational simplicity of this approach, XeF_2_ is prohibitively expensive and translation to an enantioselective, catalysis-based platform would be challenging.^61^ In 1998, Hara, Yoneda and co-workers reported the direct difluorination of alkenes using stoichiometric *p*-TolIF_2_ (**35**) and Et_3_N·HF complex.^62^ This I(iii)-reagent-based approach proceeds *via* a type II invertive mechanism (Type II_inv_), resulting in a net *syn*-addition (**34** → **36**).^58^

Inspired by this seminal study, groups led by Jacobsen^63^ and Gilmour^64^ independently developed catalytic versions of this venerable transformation. Both strategies are predicated on the oxidation of simple aryl iodide organocatalysts, in the presence of an amine·HF complex, to generate the incipient ArIF_2_ species *in situ*.^65,66^ Whilst the Gilmour protocol employed Selectfluor® and various amine : HF ratios to generate **35***in situ*, the Jacobsen method employed *m*-CPBA as the terminal oxidant in conjunction with Olah's reagent to form the resorcinol derivative **37**. Both groups disclosed preliminary validation of enantioselectivity, and this has since been expanded further to enable the generation of chiral motifs with broad functional group tolerance (*vide infra*). A scalable, electrochemical variant of the *vicinal* difluorination of alkenes mediated by *p*-TolIF_2_ has also been reported by Lennox and co-workers.^67^

**Fig. 9:**
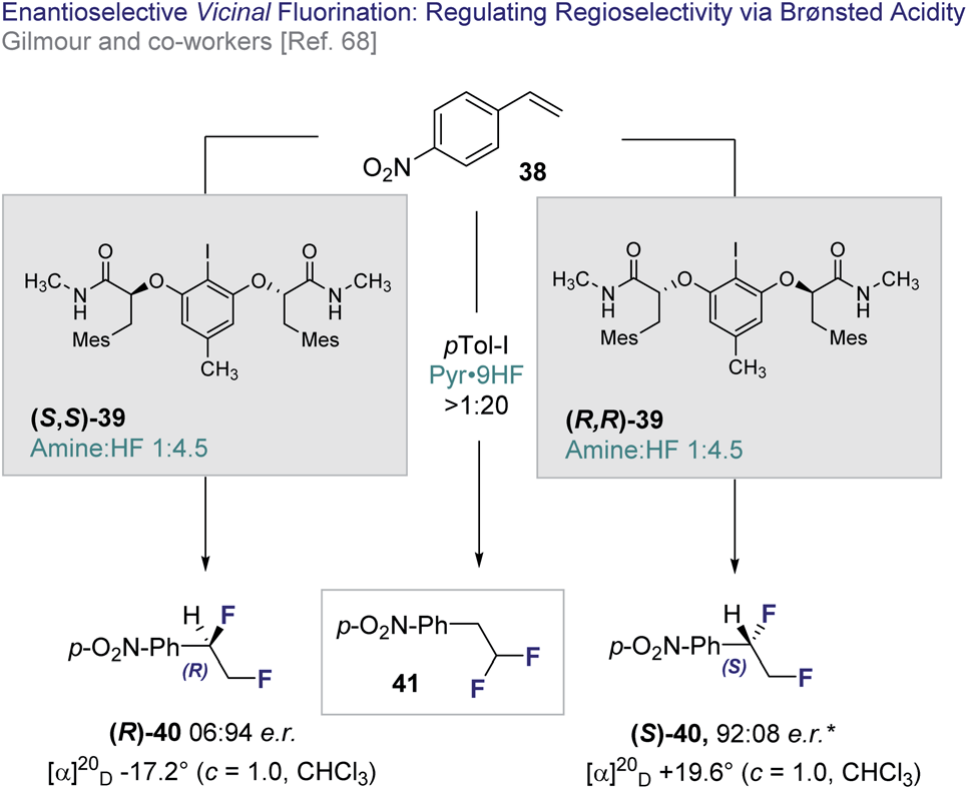
The enantioselective, catalytic *vicinal* difluorination of electron deficient styrenes. * 98 : 2 *e.r.* after recrystallisation from CH_2_Cl_2_/*n*-pentane.

In 2018, Gilmour and co-workers reported an enantioselective, catalytic *vicinal* difluorination of electron deficient styrenes (*e.g.***38**) using a chiral resorcinol-derived aryl iodide (**39**, Fig. 9).^68^ This study revealed the importance of Brønsted acidity in biasing regioselectivity (*vicinal versus geminal*, **40** and **41**, respectively) as a function of the amine : HF ratio. Varying amine : HF ratios are achieved by mixing commercially available amine·HF complexes, such as NEt_3_·3HF and Olah's reagent (Pyr·9HF). It is pertinent to note that the importance of Brønsted acid activators was reported by Cotter *et al.*^69^ in the activation of iodobenzene dichloride^23*c*,70^ by trifluoroacetic acid.

**Fig. 10:**
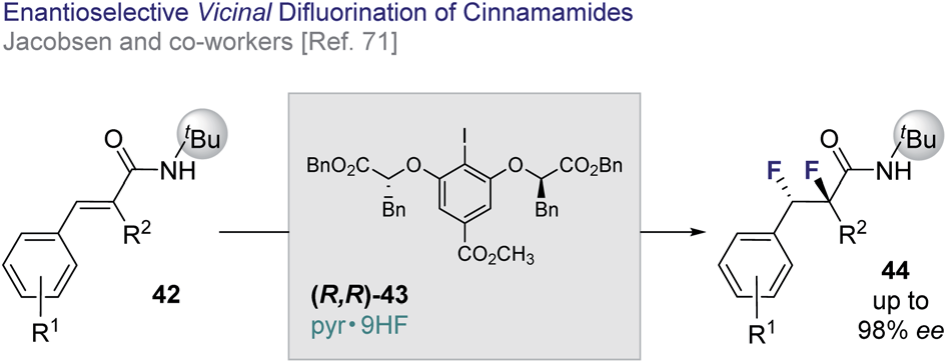
The enantio- and diastereoselective *vicinal* difluorination of cinnamamides.

Jacobsen and co-workers have reported an enantio- and diastereo-selective *vicinal* difluorination of cinnamamides (**42** → **44**) using a chiral resorcinol-based aryl iodide (**43**).^71^ Regioselectivity is regulated through the anchimeric assistance of a *N-tert*-butyl amide substituent thereby suppressing phenonium ion rearrangement to deliver the geminal product (*vide infra*). This elegant solution enables the target difluorides to be generated in up to 98% *ee* (Fig. 10).

**Fig. 11:**
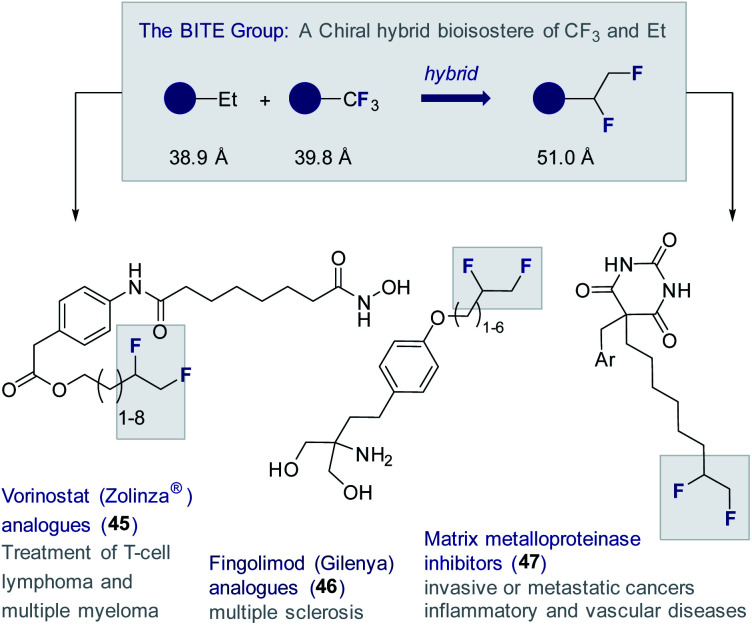
Small molecule drugs modified with the BITE group.

To date, this methodology^64^ has been leveraged to validate the 1,2-difluoromethylene motif as a chiral hybrid bioisostere of trifluoromethyl and ethyl (BITE group)^8*b*^ in several small molecule drug candidates (Fig. 11). Examples from this laboratory include the synthesis of a series of Vorinostat (Zolinza®) derivatives (**45**) containing a pendant chain capped with a *vicinal* difluoro motif.^72^ The HDAC inhibitory behaviour of this compound set was evaluated relative to the non-fluorinated systems.^73^ In all cases, the FDA approved Vorinostat (Zolinza®) was used as a control.^74^ Several of the compounds containing the 1,2-difluoroethylene unit showed greater *in vitro* potency than the clinically approved drug itself against HDAC1. This trend was found to be general with the BITE-modified HDAC inhibitors performing significantly better than the ethyl derivatives.

BITE-modified analogues of the multiple sclerosis drug Fingolimod (Gilenya®) (**46**) have also been reported.^75^ Through detailed physicochemical analyses, it was possible to demonstrate that introduction of the BITE group is accompanied by a significant reduction in lipophilicity compared to the ethyl and trifluoromethyl systems. Most recently, the BITE group has been validated as a hybrid bioisostere of the trifluoromethyl and ethyl groups using matrix metalloproteases as structural probes.^76^ To that end, a series of modified barbiturate inhibitors (**47**) were evaluated as inhibitors of MMPs 2, 8, 9 and 13.^77^ The IC_50_ values of the BITE-modified inhibitors were found to intersect those of the corresponding Et and CF_3_ derivatives.^55^

**Fig. 12:**
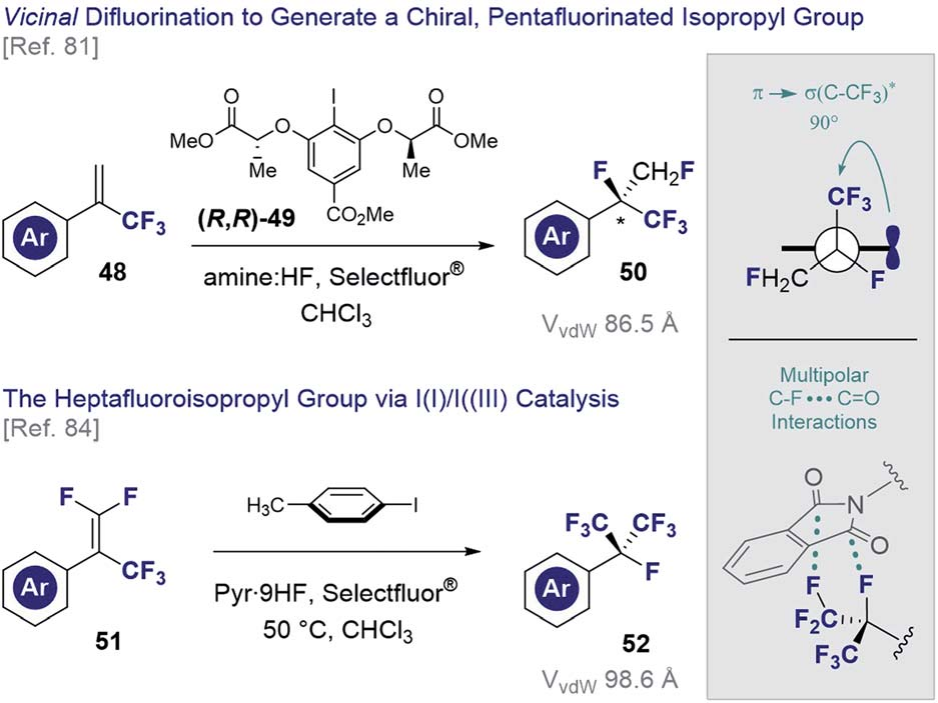
Generating fluorinated surrogates of the isopropyl group *via* I(i)/I(iii) catalysis.

The *vicinal* difluorination of alkenes has recently been extended to α-trifluoromethyl styrenes to generate fluorinated analogues of the isopropyl group (Fig. 12). Although the heptafluoroisopropyl group has become a privileged motif in agrochemical research^3,7^ and currently features in drug candidates^78^ and organocatalysts,^79^ routes to generate a chiral analogue remained conspicuously absent. Exposing simple α-trifluoromethyl styrenes (**48**) to fluorination conditions (various amine·HF complexes, Selectfluor®) in the presence of a chiral resorcinol catalyst ((*R,R*)-**49**),^80^ it was possible to generate chiral products efficiently (**50**) and with good levels of enantioselectivity.^81^ An interesting conformational feature of this motif is that the C(sp^3^)–CF_3_ bond is orthogonal to the plane of the aryl ring, thereby enabling stabilising hyperconjugative interactions,^82^ whilst mitigating 1,3-allylic strain.^83^ Moreover, the stereoelectronic *gauche* effect manifests itself as was determined by single crystal X-ray analysis of several derivatives. In an extension of this methodology, the *vicinal* difluorination of α-trifluoromethyl-β-difluoro-styrenes (**51** → **52**) was achieved through *in situ* generation of *p*-TolIF_2_ (**35**) by treatment of *p*-TolI with Selectfluor® in the presence of pyr·9HF complex.^84^ In line with the previous analysis, the structure displayed a degree of pre-organisation with one of the C(sp^3^)–CF_3_ bonds aligned with the π-system of the adjacent aryl ring. Curiously, a phthalimide derivative was found to display orthogonal C–F…C=O interactions with a neighbouring molecule in the solid state. This may prove to be useful given the increasing prominence of these interactions in medicinal chemistry.^11,85^

### 3.2 Catalysis-based *geminal* difluorination of alkenes

**Fig. 13:**
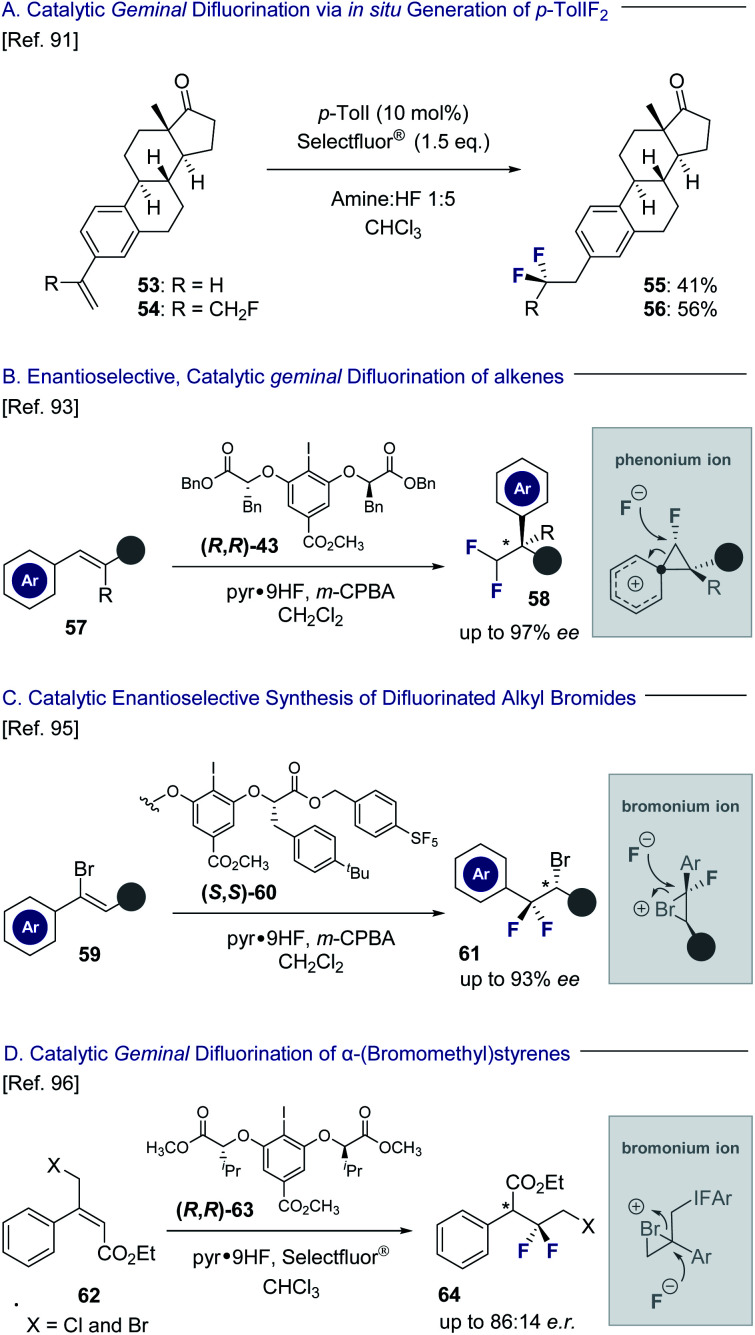
*Geminal* difluorination of alkenes to generate difluoromethylated stereocenters.

Hypervalent iodine platforms have a venerable history in halogenation chemistry,^86^ and have also been successfully harnessed to generate *geminal* difluorination patterns (Fig. 13). Seminal examples include Hara and Yoneda's use of stoichiometric quantities of *p*-TolIF_2_ (**35**) to enable a difluorinative ring contraction of alkenes.^87^ The antipodal ring expansion has recently been reported by this laboratory to generate conformationally biased fluorinated tetralins.^52^ A silver-mediated *geminal* difluorination of styrenes has been developed by Szabó and co-workers using a fluoroiodoxazole reagent.^88^ Moreover, Murphy and co-workers have disclosed the *geminal* difluorination of phenylallenes using stoichiometric *p*-TolIF_2_*via* Lewis acid activation.^89^

Catalysis-based platforms have been developed to complement these reagent-based approaches and include Kitamura and co-workers protocol to generate 2,2-difluoroethylarenes from simple styrenes using *p*-TolI as the catalyst with *m*-CPBA as the oxidant.^90^ This laboratory has also reported the *geminal* difluorination of styrenes and extended it to include α-substituted styrenes bearing fluorine-containing groups (Fig. 13A; **53** → **55** and **54** → **56**).^91^ The difluorination of alkenyl *N*-methyliminodiacetyl boronates has been reported by Fan and co-workers to generate synthetically useful building blocks for subsequent diversification.^92^ Particularly relevant to this *Perspective* dedicated to short, chiral fluorine-containing groups is the development of an enantioselective, catalytic 1,1-difluorination of alkenes (**57**) to construct difluoromethylated stereocenters (**58**) by Jacobsen and co-workers (Fig. 13B).^93^ Key to the success of this transformation is a stereospecific phenonium ion rearrangement^94^ to deliver highly versatile building blocks with excellent levels of enantioselectivity. The same laboratory has also leveraged a conceptually related reaction design, proceeding *via* bromonium ion formation, to process simple vinyl bromides to optically active difluorinated alkyl bromides (Fig. 13C, **59** → **61**).^95^ Bromonium ion formation is a feature in the *geminal* difluorination of α-(bromomethyl)-styrenes reported by this laboratory to generate electrophilic linchpins (Fig. 13D, **62** → **64**).^96^ Although the transformations discussed in Section 3.2 do not generate a stereogenic centre at the fluorine bearing carbon atom, their inclusion in this *Perspective* is instructive. Collectively, I(iii) species have been central to the development of catalysis-based methods to enable the 1,1- and 1,2-difluorination alkenes, whilst also facilitating access to 1,3-difluoro motifs.^97–99^

## 4. Conclusions

Short, alkyl groups are prominent in the natural product repertoire and are a logical consequence of the iterative biosynthesis algorithms that underpin their genesis. The importance of these seemingly inconspicuous motifs in biology is reflected in the development of many synthetic bioactive small molecules in which the “*magic methyl”* effect manifests itself. Chiral antipodes of these structural units have a venerable history in mechanistic enzymology and would augment the existing drug module portfolio. However, with the exception of branched hydrocarbons, this requires the impractical introduction of deuterium and tritium. Hydrogen to fluorine (bio)isosterism, to generate multi*vicinal* fluoroalkanes, proves an alternative to address this challenge and develop materials with unique properties. In what may be considered a conceptual merger of two units that are prevalent in nature; namely short alkyl groups and (*vicinal*) oxidation patterns, a plethora of selective processes have been reported that leverage I(i)/I(iii) catalysis to expand organofluorine chemical space into chiral regions. Integrating these fluorine-containing fragments in focussed drug and agrochemical discovery libraries will fully reveal the physicochemical potential of these materials which will, in turn, provide an impetus for further innovation in the field. In recent years, the seemingly innocent replacement of H/OH by F in stereochemically complex biomolecules has led to striking changes in orientation when bound by the target enzyme: this has broad implications for molecular recognition and chemical biology in a more general sense.^100,101^ Expanding organofluorine chemical space has an important role to play in the design of molecular function and main group catalysis is currently centre stage.

## Author contributions

The manuscript was conceived by all authors and written by RG with input from SM and JH.

## Conflicts of interest

There are no conflicts to declare.

## Supplementary Material
